# *Chlamydia muridarum* Alleviates Colitis via the IL-22/Occludin Signal Pathway

**DOI:** 10.1155/2020/8894331

**Published:** 2020-12-17

**Authors:** Xin Wang, Huai-cai Zeng, Yan-ru Huang, Qing-zhi He

**Affiliations:** ^1^School of Biotechnology, Guilin Medical University, Guilin 541199, China; ^2^Hengyang Medical School, University of South China, Hengyang 421001, China

## Abstract

Ulcerative colitis (UC) is the most common inflammatory bowel disease, and its incidence has increased in recent years. Recent clinical and experimental data indicate that gut microbiota plays a pivotal role in the pathogenesis of UC. *Chlamydia* establishes a stable and persistent colonization in the gastrointestinal tract without apparent pathogenicity to gastrointestinal or extragastrointestinal tissues. However, the detailed effects of *Chlamydia* on the gastrointestinal tissue remain unknown. The primary aim of this study is to investigate the effects of *Chlamydia muridarum* (*C. muridarum*) on development of colitis induced by dextran sodium sulfate (DSS) and the underlying molecular mechanism. The results suggested that *C. muridarum* significantly improved colitis symptoms—including weight loss, disease activity index, colon length, and histopathological changes in the colon caused by DSS—and alleviated the reduced expression of interleukin-22 and occludin in the colonic tissue due to DSS administration. Furthermore, the absence of IL-22 completely prevented *C. muridarum* from alleviating colitis and significantly decreased the levels of occludin, an important downstream effector protein of IL-22. These findings suggest that *C. muridarum* ameliorates ulcerative colitis induced by DSS via the IL-22/occludin signal pathway.

## 1. Introduction

Ulcerative colitis (UC), a type of inflammatory bowel disease, is a chronic and relapsing inflammatory disorder of the gastrointestinal (GI) tract with a rapidly increasing incidence worldwide [1]. The precise etiology and pathogenesis of UC remains unclear. It is well known that gut microbiota is involved in the pathogenesis of UC [2]. The gut microbiota regulates immune pathways, which is demonstrated by IL-22 response and barrier maintenance by epithelial tight junctions, and plays a key role in triggering UC [3].

Several studies demonstrate that gut microbiota, such as *Citrobacter rodentium* and *Escherichia coli*, enhances the intestinal barrier by promoting the expression of IL-22 and occludin [1, 4]. Gut microbiota affects intestinal inflammation by controlling the production of IL-22 [3]. IL-22, an important factor in the pathogenesis of UC as demonstrated by experiment models of colitis [5], induces the expression of tight-junction proteins (e.g., occludin and ZO-1) to facilitate gut epithelial resistance to colitis, indicating that IL-22 protects the intestinal mucosa from inflammation by upregulating the production of tight-junction proteins [6]. Occludin maintains the mucosal barrier integrity [7]. Impaired occludin, which disrupts the epithelial barrier integrity, was observed in both human UC and mouse model of colitis induced by dextran sodium sulfate (DSS) [8]. Therefore, IL-22/occludin signal pathway is important in colitis.

Chlamydial organisms can be detected in the GI tracts of some animals and humans [99. *Chlamydia* inoculated into mouse GI tract readily colonized the GI tract and persisted for long periods [10]. Interestingly, the long-lasting GI tract infection by *Chlamydia* did not cause significant inflammatory pathology in the host [11]. However, the medical significance of *Chlamydia* in the GI tract remains unclear. Exploring the potential functions of *Chlamydia* in the GI tract may provide useful information about *Chlamydia* infections. We will investigate the therapeutic effect of *Chlamydia muridarum* in the GI tract on colitis and its underlying mechanisms in C57BL/6J mice treated with DSS.

## 2. Materials and Methods

### 2.1. *Chlamydia* Culture

All *Chlamydia muridarum* clones used in the present study were derived from strain Nigg3 (GenBank accession number CP009760.1), including a passaged clone designated G13.32.1 [12], provided by Dr. Zhong at the University of Texas Health Science Center (San Antonio, United States). G13.32.1 was propagated in HeLa cells and purified as elementary bodies (EBs), as reported previously [13]. Aliquots of the purified EBs were stored at -80°C until use.

### 2.2. Mouse Infection

The mouse experiments were performed in accordance with the recommendations in the guide for the experimental animal ethics committee of the University of South China. C57BL/6J female mice (5–6-week-old; Experimental Animal Center of the University of South China, Hengyang, China) were intragastrically inoculated with purified *C. muridarum* EBs using 2 × 10^5^ inclusion-forming units in sucrose-phosphate-glutamic acid (SPG) medium (220 mM sucrose, 12.5 mM phosphate, and 4 mM L-glutamic acid; pH 7.5), as described previously [14]. Live *C. muridarum* EBs suspended in 200 *μ*l of SPG buffer were delivered to each mouse using a straight ball-end needle (item number N-PK 020; Braintree Scientific, Inc., Braintree, MA).

### 2.3. Experimental Design

Mice were bred in a pathogen-free, temperature-controlled environment on a 12 h light and dark cycle at the Animal Center of the University of South China. Mice were divided randomly into four groups: control, DSS, CM+DSS, and CM+DSS+aIL-22. The control group mice (untreated healthy mice) received fresh, sterile, distilled water. The mice in the DSS, CM+DSS, and CM+DSS+aIL-22 groups received 3% (*w*/*v*) DSS (36000–50000 MW, MP Biomedicals, Solon, OH, USA) in drinking water for 5 days (day 1 to days 5; fresh DSS solution prepared every 2 days) to induce an acute episode of colitis from the 28 days postchlamydial inoculation. Moreover, the CM+DSS+aIL-22 group mice were fed with DSS and simultaneously received an intraperitoneal injection of 100 *μ*g anti-IL-22 (eBioscience, catalog 16-7222-82) for 5 days.

### 2.4. Immunofluorescence Assay

For monitoring of live organism shedding, rectal swabs were taken every 3 to 4 days for the first week and weekly thereafter. To quantitate live chlamydia, each swab was soaked in 0.5 ml of SPG buffer and vortexed with glass beads, and the chlamydial organisms released into the supernatants were titrated on HeLa cells. The infected cultures were processed for immunofluorescence assay as described previously [15]. A rabbit antibody (designated R1604, raised against purified *C. muridarum* EBs) was used as a primary antibody to label *C. muridarum* in HeLa cells and was then visualized with goat anti-rabbit IgG conjugated with Cy2 (green; Solarbio, SE131). The DNA dye Hoechst 3328 (blue; Solarbio) was used to visualize the nuclei. The doubly labeled samples were used to count *C. muridarum* bacteria under a fluorescence microscope (model DMi8; Leica, Germany) equipped with a charge-coupled device camera (M205FA; Leica, Germany).

### 2.5. Disease Activity Index

Weight loss, bleeding, and diarrhea in mice were assessed daily and recorded since day 1 before DSS administration; (a) weight loss: 0 = none, 1 = 1-5%, 2 = 5-10%, 3 =10-15%, and 4 = over 15%; (b) stool consistency: 0 = normal stool, 2 = loose or pasty pellets, and 4 = diarrhea; and (c) blood in stool: 0 = normal, 2 = positive occult blood, and 4 = rectal bleeding. The mean (*m*) of all scores was calculated and recorded as disease activity index (DAI) as described previously [16].

### 2.6. Colon Measurement

At the end of the experiment, the mice were euthanized by cervical dislocation under ether narcotization. Their colons (from the end of the cecum to the beginning of the rectum) were removed and photographed, and the length of the colon was measured.

### 2.7. Histological Analysis

Dissected colon tissues were immediately immersed in 4% paraformaldehyde (Sigma-Aldrich, P6148). The tissue samples were processed in molten paraffin in cassettes and kept at -20°C until paraffin solidified completely. Paraffin blocks were trimmed and then sectioned into 4 *μ*m sections using a microtome (Leica, SP1600). The sections were picked up with a paintbrush, placed on the surface of deionized water in a bath at 40°C, and transferred onto histological slides. The slides were oven-dried (60°C) and dehydrated with different concentrations of alcohol (Sinopharm, 100092683), and samples were stained with hematoxylin solution (Servicebio, G1005) for 5 min and differentiated with acid alcohol (Sinopharm, 100092683). After washing the slides under slowly running tap water, the samples were stained with eosin (Servicebio, G1005) for 5 min, dehydrated with alcohol (Sinopharm, 100092683), and then photographed under a light microscope (Olympus, BX53).

### 2.8. Western Blot Analysis

The colon samples were washed twice with cold phosphate-buffered saline; 1 ml of ice-cold RIPA buffer (Servicebio, catalog: G2002) was added per 100 mg tissue. Protease inhibitor PMSF (100 mM, Servicebio, G2008) was added before use. Then, the colon tissue was homogenized with an electric homogenizer (KangTao Biological Technology, KZ-II). Homogenized tissue was kept for 30 min in an ice bath and constantly mixed using a pipette; the mixture was then centrifuged for 10 min at 12,000 rpm at 4°C in a microcentrifuge (Heal Force, Neofuge 13R). The supernatant was gently aspirated and placed in a fresh tube kept on ice. The total protein concentration was determined using the BCA kit (Servicebio, G2026). Separating and loading gel buffers for SDS-PAGE were prepared according to the manufacturer's instructions (Servicebio, G2003). Appropriate quantities of protein samples and molecular weight marker were loaded into the wells of the SDS-PAGE gel, and electrophoresis was performed for 1.5 h (75 V for 30 min in the stacking gel and 120 V for 1 h in the separating gel). The protein was transferred from the gel to a 0.22 *μ*m PVDF membrane (Millipore, ISEQ00010; the membrane was blocked using 5% milk buffer (Servicebio, G5002) for 1 h at room temperature and then incubated overnight at 4°C with primary antibodies against rabbit IL-22 (1 : 1,000, Affinity Biosciences, LBP70748), occludin (1 : 2,000, Affinity Biosciences, 4970 T), and *β*-actin (1 : 2,000, Affinity Biosciences, SE131). Next, the membrane was incubated with goat anti-rabbit IgG secondary antibodies (1 : 4,000, Affinity Biosciences, OH, USA) at room temperature for 1 h. An enhanced chemiluminescence (ECL; Servicebio, G2014) western blot was performed, and an image was acquired as described by the manufacturer. Alpha processing system (Alpha Innotech, alpha EaseFC) was used to analyze the optical density value of the target band. *β*-Actin was used as an internal control.

### 2.9. Statistical Analysis

All data, including the time course of live organism shedding measured as the number of inclusion-forming units, body weight loss, DAI, colon length, and relative protein expression, were compared using one-way ANOVA.

## 3. Results

### 3.1. *Chlamydia muridarum* Colonized the Gastrointestinal Tract Stably and for Extended Periods

To determine whether *C. muridarum* can colonize the gastrointestinal tract of female mice stably and for long periods, we monitored the growth of *C. muridarum* in the GI tract of intragastrically inoculated mice using immunofluorescence assays (Table 1). *C. muridarum* was detected on day 3 after intragastrical inoculation and increased on day 7. *Chlamydia muridarum* colonized stably in the GI tract from day 7 to day 28. *Chlamydia muridarum* growth did not differ significantly between groups CM+DSS and CM+DSS+aIL-22. Thus, *C. muridarum* establishes a stable and persistent colonization in the GI tract. This finding made it possible to conduct the next experiment.

### 3.2. Effect of *Chlamydia muridarum* on Mouse Body Weight and Disease Activity Index Induced by Dextran Sodium Sulfate

**Figure 1:**
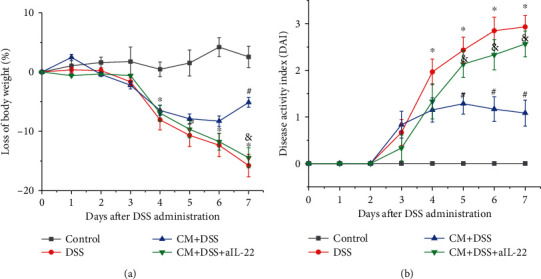
*Chlamydia muridarum* ameliorated the mice body weight loss and the disease activity index induced by DSS. (a) Loss of body weight. (b) Disease activity index. The data were evaluated as mean ± SD (*n* = 10 mice per group). ^∗^*P* < 0.05, DSS group compared with control group; ^#^*P* < 0.05, CM+DSS group compared with the DSS group; ^&^*P* < 0.05, CM+DSS+aIL-22 group compared with CM+DSS group.

**Figure 2:**
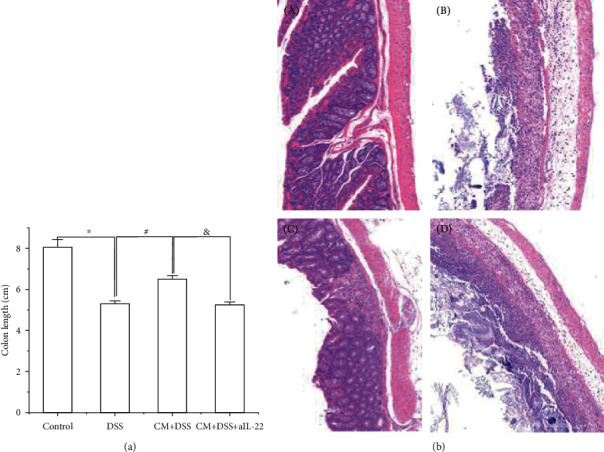
Effect of *Chlamydia muridarum* on colitis induced by dextran sodium sulfate (DSS). (a) colon length; the data were evaluated as mean ± SD (*n* = 10 mice per group). (b) Representative photographs of colon sections with hematoxylin and eosin staining. (a) Control group. (b) DSS group. (c) CM+DSS group. (d) CM+DSS+aIL22. ^∗^*P* < 0.05, DSS group compared with control group; ^#^*P* < 0.05, CM+DSS group compared with DSS group; ^&^*P* < 0.05, CM+DSS+aIL-22 group compared with CM+DSS group.

As shown in Figure 1, the body weight of control mice remained unchanged, whereas the mice treated with DSS began to lose weight significantly from Day 4 after DSS treatment. Infection with *C. muridarum* prevented the weight loss and promoted weight regain from day 6. The body weight loss of CM+DSS+aIL-22 mice was higher than that of CM+DSS mice on day 7. There was no statistical difference between body weight loss in the DSS and CM+DSS+aIL-22 groups (Figure 1(a)). The DAI of the DSS group mice on days 4, 5, 6, and 7 was 1.96, 2.43, 2.85, and 2.93 units higher than that of the control group mice, respectively. The DAI of the CM+DSS group mice for days 5, 6, and 7 was 1.28, 1.16, and 1.08 units lower than that of DSS group mice, respectively. The DAI of the CM+DSS+aIL-22 group mice for days 5, 6, and 7 were 2.13, 2.33, and 2.57 units higher than that of CM+DSS group mice, respectively (Figure 2(b)).

### 3.3. *Chlamydia muridarum* Alleviates Colitis Induced by Dextran Sodium Sulfate

As shown in Figure 2, the colon length of DSS mice was lower than that of control mice. *Chlamydia muridarum* alleviated the damage to colon length induced by DSS, and aIL-22 prevented the effect of *C. muridarum* on the colon length. Therefore, there was no significant difference between DSS and DSS+CM+aIL-22 groups. Hematoxylin and eosin staining results showed that control mice had clear colon structures (Figure 2(b), A). The submucosa and muscular layer were normal. The DSS group mice suffered from severe ulcers (Figure 2(b), B). The mucous epithelial layer was completely defective, and all recesses were destroyed, accompanied by significant infiltration of inflammatory cells, mainly macrophages and neutrophils. The injury invaded the submucosa and muscle layer. The submucosa showed severe edema, dilated blood vessels, and inflammatory cell infiltration. The muscle fibers of the muscle layer showed necrosis. The CM+DSS group mice had basically normal colon structure, and focal inflammatory cell (mainly lymphocyte) infiltration was occasionally found in the mucous layer (Figure 2(b), C). The mice in the CM+DSS+aIL-22 group showed colitis similar to those in the DSS group (Figure 2(b), D). The colon ulcers were severe, and the mucous epithelial layer was completely defective. The intestinal gland disappeared, and significant plasma cell and neutrophil infiltration was observed. The injury invaded the submucosa and muscle layer. The submucosa showed edema and scattered inflammatory cell infiltration.

### 3.4. *Chlamydia muridarum* Alleviates Colitis Induced by Dextran Sodium Sulfate via the IL-22/Occludin Signal Pathway

**Figure 3:**
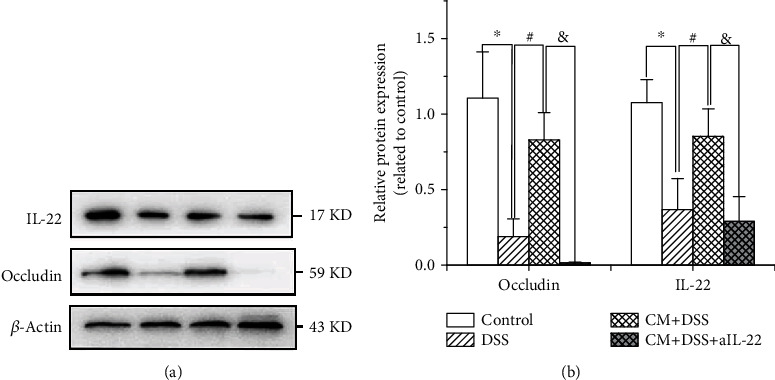
*Chlamydia muridarum* upregulates IL-22 and occludin expression in mice with dextran sodium sulfate- (DSS-) induced colitis. (a) IL-22 and occludin expression in various groups assessed using western blotting. (b) Relative protein expression of IL-22 and occludin. (*n* = 3 per group). ^∗^*P* < 0.05, DSS group compared with control group; ^#^*P* < 0.05, CM+DSS group compared with DSS group; ^&^*P* < 0.05, CM+DSS+aIL-22 group compared with CM+DSS group.

We further investigated the mechanism by which *C. muridarum* alleviates colitis induced by DSS. As shown in Figure 3, western blotting showed that treatment by DSS downregulated IL-22 and occludin. *Chlamydia muridarum* significantly alleviated the IL-22 and occludin downregulation induced by DSS. When mice were treated with aIL-22, the absence of IL-22 significantly inhibited the effect of *C. muridarum* on IL-22 and occludin expression. No significant difference was found in IL-22 and occludin levels between DSS group and DSS+CM+aIL-22 group. Taken together, these findings suggest that *C. muridarum* upregulates IL-22 and occludin in mice with DSS-induced colitis. Therefore, we conclude that *C. muridarum* alleviates colitis induced by DSS via the IL-22/occludin signal pathway.

## 4. Discussion

Since the late 1990s, *Chlamydia* has been the most commonly reported sexually transmitted infection [13]. *Chlamydia trachomatis* is a sexually transmitted bacterial pathogen that causes clinical pathology of the upper genital tract [17]. It has been hypothesized that long-term chlamydial colonization of the GI tract serves as a reservoir for autoinoculation of the genital tract [18]. Although chlamydial organisms have been detected in the GI tracts of both humans and animals and *C. muridarum* is known to colonize the GI tract for long periods, the medical significance of these phenomena remains unclear [19]. *Chlamydia muridarum* is a model pathogen to investigate the pathogenesis of *C. trachomatis*, which spreads readily from the genital tract to the gastrointestinal tract of mice and then establishes a persistent colonization [11]. Our experiments demonstrated that *C. muridarum* establishes a persistent, stable colonization in the gastrointestinal tract of intragastrically inoculated mice, which is consistent with previous reports. Despite robust specific immunoglobulin A (IgA) responses in the gut, they remain in the gut for long periods, similar to other commensal microbial species [9]. We speculate that *C. muridarum* has established itself as a normal commensal in the cecal and colonic epithelial tissues.

Ulcerative colitis is a multifactorial disease of unclear etiology, which arises as a result of the interaction of genetic, environmental, barrier, and microbial factors leading to immunological responses and chronic inflammation in the intestine [20]. The intestinal epithelial cells, mucus layer, antimicrobial peptides, and microbe-specific IgA synthesized in the Peyer's patches (PPs) constitute the mucosal firewall [21]. It acts as a selective barrier to limit the entry of antigens into the mucosal immune system and induce oral tolerance to commensal microorganisms/food antigens or host defense against pathogens. Epithelial tight junctions allow for the selective penetration of nutrients, fluids, and microorganisms [22]. It is reported that gut microbiota affects the intestinal immune barrier and limits microbial translocation [21]. For example, *E. coli* flagellin enhances TLR5 signaling in intestinal DCs, leading to a higher expression of IL-22 and better maintenance of the intestinal barrier, to protect mice against DSS-induced colitis [23]. Our research showed that *C. muridarum* effectively attenuated DSS-induced UC. Many lactic acid bacteria strains can effectively resist enteritis by adhering to intestinal mucosa and producing antimicrobial compounds against pathogens [24]. Therefore, we speculate that *Chlamydia* also improves UC by improving the gastrointestinal barrier.

Recent studies suggest that IL-22 prevents tissue damage caused by inflammation and infection, promotes wound healing, and restores the mucosal barrier integrity of the intestine tissue [25]. IL-22, a T-helper 17 (Th17) cell-associated cytokine, promotes cell proliferation and tissue regeneration to protect intestine tissues [26] and regulates UC through multiple pathways including cytokine response pathways and by promoting cell proliferation and strengthening tight junctions [23, 27–29]. Moreover, IL-22-deficient mice exhibit a significantly increased susceptibility to enteric pathogen infection and experimental colitis. During HIV chronic infection, IL-22 derived from mucosal lymphocytes is depleted, and the gut epithelial integrity is destroyed [30]. In addition, IL-22 upregulates the expression of occludin to maintain the gut epithelial barrier [28].

Occludin is an important intercellular tight-junction protein associated with mucosal barrier and plays a central role in intestinal functions [31]. It is also a major determinant of transepithelial transport and mucosal permeability. The absence of occludin leads to impaired integrity of the intestinal epithelial barrier [32]. Many studies in vitro on occludin gene knockdown [33–35], occludin gene mutants [36], and peptides corresponding to occludin domains [37] suggest that occludin limit macromolecules to transepithelial transport and maintain the mucosal barrier integrity. Occludin participates in the cellular mucosal barrier as it polymerizes on the plasma membrane surface and completely circumscribes the apex of cells. Instead of assembling and disassembling the whole tight junction complex, the tight junction permeability barrier can be regulated by recruiting and removing occludin to and from the tight junction, respectively [38]. It has been demonstrated that, in mice, osteopontin protects colonic mucosa from DSS-induced acute colitis by regulating occludin [39]. End1/E6E7 cells treated with IL-22 produce higher levels of ZO-1 and occludin than untreated cells [40]. In UC, treatment with IL-22 could protect the intestinal mucosa from inflammation via increased production of occludin [31, 41]. Therefore, IL-22/occludin signal pathway plays an important role in colitis.

To demonstrate that C. muridarum may alleviate DSS-induced colitis via the IL-22/occludin signaling pathway, we used an anti-IL-22 antibody to determine the specific role of IL-22 and occludin. Our findings show that occludin is an important effector of IL-22 in maintaining the intestinal barrier integrity and *C. muridarum* can upregulate IL-22 and occludin expression in DSS-induced mice, which suggests that *C. muridarum* alleviate DSS-induced UC via IL-22/occludin signal pathway. It will provide notable insights into the molecular and cellular bases of chlamydial interactions with the GI tract and offers significant information for drug development for colitis and Chlamydia oral vaccines.

## Figures and Tables

**Table 1:** *Chlamydia muridarum* in the gastrointestinal tract (log_10_($\bar{\text{x}}\pm \text{s}$)).

Group	Day 3	Day 7	Day 14	Day 21	Day 28
CM+DSS+aIL-22	0.81 ± 0.73	3.42 ± 1.00	3.31 ± 0.75	3.61 ± 1.15	3.64 ± 0.81
CM+DSS	0.76 ± 0.95	3.60 ± 1.20	3.51 ± 0.60	3.31 ± 1.25	3.68 ± 0.72
DSS	ND	ND	ND	ND	ND
Control	ND	ND	ND	ND	ND

(ND: undetected). Days after inoculation.

## Data Availability

The data used to support the findings of this study are included within the article.
